# Estrogens Regulate the Hepatic Effects of Growth Hormone, a Hormonal Interplay with Multiple Fates

**DOI:** 10.3389/fendo.2013.00066

**Published:** 2013-06-03

**Authors:** Leandro Fernández-Pérez, Borja Guerra, Juan C. Díaz-Chico, A. Flores-Morales

**Affiliations:** ^1^Oncology-Molecular and Translational Endocrinology Group, Clinical Sciences Department, Faculty of Health Sciences, Associate Unit of University of Las Palmas de Gran Canaria and Biomedical Institute “Alberto Sols”-CSIC, Las Palmas de Gran Canaria, Spain; ^2^Molecular Endocrinology Group, Novo Nordisk Center for Protein Research, University of Copenhagen, Copenhagen, Denmark

**Keywords:** growth hormone, estrogen, liver, metabolism, sexual dimorphism

## Abstract

The liver responds to estrogens and growth hormone (GH) which are critical regulators of body growth, gender-related hepatic functions, and intermediate metabolism. The effects of estrogens on liver can be direct, through the direct actions of hepatic ER, or indirect, which include the crosstalk with endocrine, metabolic, and sex-differentiated functions of GH. Most previous studies have been focused on the influence of estrogens on pituitary GH secretion, which has a great impact on hepatic transcriptional regulation. However, there is strong evidence that estrogens can influence the GH-regulated endocrine and metabolic functions in the human liver by acting at the level of GHR-STAT5 signaling pathway. This crosstalk is relevant because the widespread exposition of estrogen or estrogen-related compounds in human. Therefore, GH or estrogen signaling deficiency as well as the influence of estrogens on GH biology can cause a dramatic impact in liver physiology during mammalian development and in adulthood. In this review, we will summarize the current status of the influence of estrogen on GH actions in liver. A better understanding of estrogen-GH interplay in liver will lead to improved therapy of children with growth disorders and of adults with GH deficiency.

## Introduction

Growth hormone (GH) is the main regulator of somatic growth, metabolism, and gender dimorphism in liver (LeRoith and Yakar, 2007; Lichanska and Waters, 2008; Baik et al., 2011; List et al., 2011). GH is predominantly linked to linear growth during childhood, but continues to have important metabolic actions throughout life. GH deficiency in adulthood causes a metabolic syndrome-like phenotype (i.e., increased adiposity, decreased muscle mass, metabolic disturbances, increased vascular complications) (Loria et al., 2009). The absence of estrogen signaling causes similar metabolic phenotype (Barros and Gustafsson, 2011). This metabolic syndrome-like phenotype can be ameliorated by GH (LeRoith and Yakar, 2007) or 17β-estradiol (E2) (Heine et al., 2000; Simpson et al., 2005; Jones et al., 2007) replacement which suggests that GH and E2 regulate overlapping cellular networks.

17β-Estradiol, a major natural estrogen in mammals, has physiological actions which are not limited to reproductive organs in both females and males (Simpson et al., 2005; Barros and Gustafsson, 2011). The liver is a direct target of estrogens because it expresses estrogen receptor alpha (ERα) which is connected, among others, with lipid and glucose homeostasis (Foryst-Ludwig and Kintscher, 2010; Barros and Gustafsson, 2011; Faulds et al., 2012) and body growth (Vidal et al., 2000). Estrogens can modulate GH actions in liver by acting centrally, regulating pituitary GH secretion, and, peripherally, modulating GH signaling. Most previous studies have been focused on the influence of estrogens on pituitary GH secretion (Kerrigan and Rogol, 1992). The sex-specific GH secretion release from pituitary has been shown to have a great impact on hepatic transcriptional regulation (Mode and Gustafsson, 2006). However, there is also strong evidence that estrogens modulate GH action at the level of GHR expression and signaling. Particularly, E2 has been shown to induce suppressor of cytokine signaling (SOCS)-2 which in turn negatively regulates GHR-Janus kinase (JAK)-2-signal transducer and activator of transcription (STAT)-5 signaling pathway (Leung et al., 2004; Santana-Farre et al., 2008). This phenomenon is clinically relevant because the GHR-JAK2-STAT-5 signaling is of particular importance in the regulation of endocrine, metabolic, and sex-differentiated actions of GH in liver. Importantly, disruption of GHR-JAK2-STAT5 signaling is associated with hepatic metabolic changes that include fatty liver, fibrosis, and hepatocellular carcinoma (Baik et al., 2011). This interplay is also relevant because the widespread exposition of estrogen or estrogen-related compounds in human (Wolthers et al., 2001). In this work, we will summarize the multiple biological consequences that can appear after the interplay of E2 with GH in liver. A better understanding of estrogen-GH interplay will lead to improved therapy of children with growth disorders and of adults with GH deficiency.

## Physiological Basis of Pituitary GH Secretion

**Figure 1:**
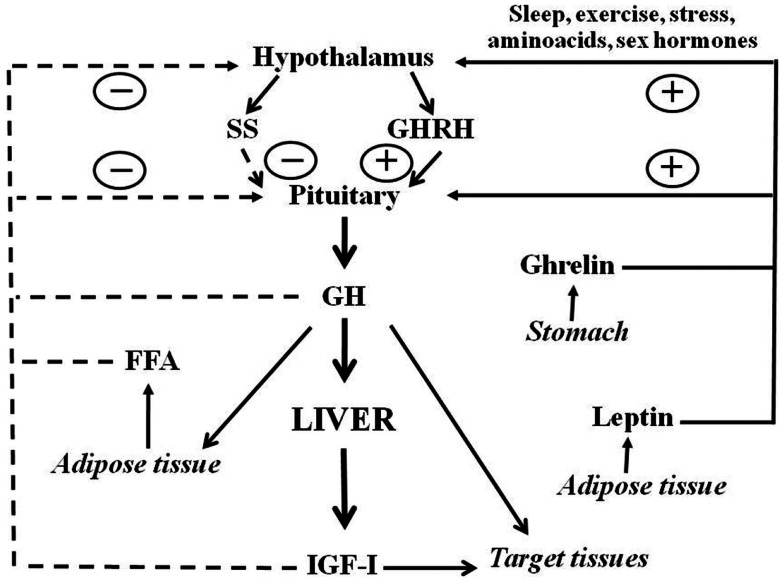
**Schematic representation of somatotropic axis**. GHRH and SS, two hypothalamic hormones, control the synthesis and release of GH from the pituitary. GHRH is negatively (dashed lines) regulated by feedback from blood GH and IGF-I concentrations. FFA also inhibits whereas leptin and ghrelin stimulate GH release. Sex hormones and other factors also act centrally to stimulate GH release. Circulating GH acts directly on many organs to stimulate IGF-I production, with IGF-I production in the liver providing the main source of blood IGF-I. GH also has direct effects on many target tissues which can be independent of IGF-I action.

Growth hormone is a polypeptide mainly secreted from the somatotrophs within the anterior pituitary gland. In addition to the pituitary, GH is produced in extra-pituitary tissues, which indicates that GH has local paracrine-autocrine effects, distinct from its classic endocrine somatotropic effects (Waters et al., 1999). The regulation of pituitary GH secretion involves a complex neuroendocrine control system that includes the participation of several neurotransmitters and the feedback of hormonal and peripheral (metabolic) factors (Butler and Le Roith, 2001; Kaplan and Cohen, 2007). Figure 1 shows that GH secretion from pituitary gland is regulated by two major hypothalamic peptides: the stimulatory GH releasing hormone (GHRH) and the inhibitory hormone somatostatin (SS). The balance of these stimulating and inhibiting peptides is in turn, indirectly, affected by many physiological stimulators (i.e., sex hormones, nutrients, sleep, and exercise) and inhibitors [i.e., insulin growth factor I (IGF-I), and GH]. In addition to hypothalamic (GHRS, SS) and endocrine (IGF-I, GH) factors, other peripheral (metabolic) factors influence pituitary GH release: free fatty acids (FFA), insulin, glucose, amino acids, leptin, neuropeptide Y, and ghrelin. These factors are primarily related to or derived from the metabolic status of the organism, which is consistent with the role of GH in regulating substrate metabolism, adiposity, and growth, and appear to coordinate the metabolic status of the organism with GH secretion (Howard et al., 1996; Carro et al., 1997; Svensson et al., 1998; Holst and Schwartz, 2006). Sex steroids are also physiological regulators of pituitary GH secretion. Both neonatal and post-pubertal sex steroids control the ability of the hypothalamus to drive the sexually dimorphism of pituitary GH secretion in adulthood (Kerrigan and Rogol, 1992). Sexual dimorphism in rodents seems to be regulated by estrogen secretion in adult females and by androgen secretion, neonatally and during adulthood, in males. Neonatal exposure to testosterone imprints the male program of neuroendocrine control of the pulsatile pituitary GH secretion that is first seen at puberty, when the adult pattern of GH secretion becomes evident, and continues throughout adulthood. If such an androgen re-programing does not occur, the secretion pattern will remain as the feminine pattern (continuous GH secretion) (Mode and Gustafsson, 2006). The sexually dimorphic pattern of GH secretion is also seen in humans, but not as marked as in the rat. Interestingly, depletion of liver-derived IGF-I in male mice (i.e., the LID mice) causes a feminization of some of the GH-regulated sexually dimorphic markers of liver functions (Ohlsson et al., 2009). This is a consequence of losing the feedback effect exerted by IGF-I on the hypothalamic-pituitary system which results in increased GH secretion, including elevated baseline GH levels between pulses, which resemble a female pattern of pituitary GH release.

## The Cellular Regulation of GH Signaling

**Figure 2:**
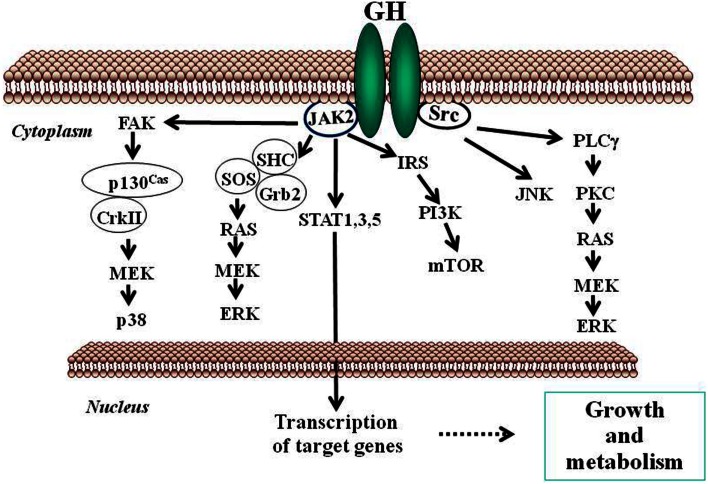
**Schematic representation of signaling pathways used by GH to regulate growth and metabolism**. GH binds to a preformed GHR dimmer which results in activation of JAK2 tyrosine kinase bound to the receptor box 1 sequence proximal to the membrane. Simultaneously, Src kinase is also activated. Canonical protein-tyrosine kinase JAK2 signaling via STAT5 involves phosphorylation of key tyrosine residues in the cytoplasmic domain of GHR, which bind the Src homology 2 (SH2) domain of STAT5a/b, recruiting STATs to the activated JAK2 and thus facilitating their tyrosine phosphorylation and subsequent dimerization through their SH2 motifs. Dimerized STAT5 translocates to the nucleus to regulate gene transcription. STAT1 and STAT3 undergo direct tyrosine phosphorylation by JAK2 without the requirement for receptor binding. ERK can be activated either by SRC and/or PLCγ and Ras, or by JAK2 via the adaptors SHC, GRB, and SOS. JNK is activated by SRC. The PtdIns 3-kinase and the serine-threonine-protein kinase mTOR pathway is activated by JAK2 via IRS phosphorylation. These signaling pathways influence, directly or indirectly, transcription of genes involved in growth and metabolism. ERK, extracellular signal-regulated kinase; FAK, focal adhesion kinase; Grb, growth factor receptor-bound protein; IRS, insulin receptor substrate; JAK2, Janus kinase 2; JNK, c-Jun N-terminal kinase; MEK, dual specificity mitogen-activated protein kinase kinase 2; mTOR, mammalian target of rapamycin; PI3K, phosphoinositide 3-kinase; PKC, protein kinase C; PLCγ, phospholipase Cγ; SHC, SH2-domain containing transforming protein; SOCS, suppressor of cytokine signaling; SOS, son of sevenless; SRC, proto-oncogene tyrosine-protein kinase Src; STAT, signal transducer and activator of transcription.

Growth hormone mediates its intracellular effects via the GHR which is ubiquitously expressed, especially in liver, fat, and muscle. GHR belongs to type I cytokine receptor, a family of receptors without intrinsic kinase activity (Brooks et al., 2008). GHR has an extracellular domain which is connected to a cytoplasmic domain via a flexible linker. The kinase JAK2 is constitutively associated with a Box 1 region in the cytoplasmic domain of the GHR. In the inactive state, the JAK2 catalytic domain is masked by its pseudokinase domain. The GH molecule interacts with preformed dimmers of identical GHR pairs, which results in a conformational change in the receptors and associated JAK2 molecules (Brown et al., 2005). This event unmasks the catalytic domain of JAK2 and results in activation of adjacent JAK2 molecules by transphosphorylation. Activated JAK2 phosphorylates the GHR cytoplasmic domain on tyrosine residues and subsequent JAK2-dependent and -independent intracellular signal transduction pathways evoke pleiotropic cell responses including changes in gene transcription, cell proliferation, glucose and lipid metabolism, or in cytoskeletal re-organization (Flores-Morales et al., 2001; Rico-Bautista et al., 2004; Tollet-Egnell et al., 2004; Vijayakumar et al., 2010). The main event in the GH signaling pathway is the recruitment of members of the STAT family of transcription factors to phosphorylated tyrosine residues in the GHR intracellular domain. Of the various STAT proteins (STAT 1–6), STAT5b has been widely associated with GH biological actions in liver (Waxman and O’Connor, 2006; Baik et al., 2011); although STAT1, 3, and 5a have also been shown to be recruited by the GHR. STAT5 phosphorylation by JAK2 results in their dissociation from the receptor, dimerization, and translocation to the nucleus where they modulate the transcription of target genes [e.g., IGF-I, acid labile subunit (ALS), SOCS2, SOCS3, CIS] (Rowland et al., 2005; Vidal et al., 2007). The STATs represent one of known pathways in GH-induced signaling; others include the MAPK and PI3K pathways (Figure 2).

The analysis of molecular mechanisms involved in inactivation of GHR-signaling pathways is also imperative for understanding GH physiology. The duration of GH-activated signals is a critical component in relation to the biological actions of this hormone. This is clearly illustrated in the case of hepatic GH actions where signal duration regulates gender differences in liver gene expression (Waxman and O’Connor, 2006). The male pattern of pituitary GH secretion in rats is episodic with peaks every 3–4 h and no measurable trough levels (Mode and Gustafsson, 2006). Consequently, intracellular activation of STAT5 is also episodic and periods with low GH circulating levels are required to achieve maximal activation of STAT5. Female rats, which exhibit a more continuous GH secretion pattern with higher basal levels and smaller and irregular intermittent peaks show reduced STAT5b activation compared with males (Mode and Gustafsson, 2006). These differences in STAT5b activation are responsible for several of the gender differences in hepatic gene expression. The conserved control of GHR-JAK2 activation kinetic in multiple cell models emphasizes the importance of mechanisms for desensitization of GH-dependent signaling pathway in GH physiology (Flores-Morales et al., 2006). Studies on primary hepatocytes and several cell lines have shown that GH-induced JAK2-STAT5b activation is transient, with maximal activation achieved within the first 30 min of stimulation, followed by a period of inactivation. This period is characterized by an inability to achieve maximal JAK2-STAT5 activation by GH in the following 3–4 h, unless GH is withdrawn from the media (Fernández et al., 1998). Cell-surface levels of GHR are the primary determinant of GH responsiveness. Transcriptional, translational, and posttranslational level factors can influence GHR synthesis and, thereby, regulate cell sensitivity to GH actions. These factors include nutritional status, endocrine context, developmental stage, and, relevant to this review, estrogens. GHR cell-surface translocation is also directly inhibited by IGF-I, likely contributing to a local feedback loop to hamper GH sensitivity (Leung et al., 1997). Removal of cell-surface GHRs by endocytosis is an early step in the termination of GH-dependent signaling. GHR ubiquitination is a key control mechanism in the down-regulation of GH signaling, modulating both GHR internalization and proteasomal degradation. The ubiquitin ligase SOCS2 has been shown to be a key components of negative regulators of GHR-JAK2-STAT5 signaling pathway (Rico-Bautista et al., 2006; Vesterlund et al., 2011). SOCS proteins have been shown to modify cytokine actions through a classic negative feedback loop. In general, SOCS protein levels are constitutively low, but their expression is rapidly induced by stimulation with different cytokines including GH. Evidence also indicates that growth factors (e.g., insulin), xenobiotics (e.g., dioxin, statins), and estrogens, can induce SOCS2 expression (Rico-Bautista et al., 2006; Santana-Farre et al., 2008). Consequently, regulation of SOCS2 protein expression provides a mechanism for crosstalk where multiple factors, including estrogens can regulate the activity of GH (Leung et al., 2004). SOCS2 binds the GHR complex and promote its ubiquitination and subsequent proteasomal degradation (Vesterlund et al., 2011). The phenotype of SOCS2 null mice (SOCS2KO) identifies SOCS2 as the key physiological player in the negative regulation of GH-dependent body growth (Horvat and Medrano, 2001; Greenhalgh et al., 2005; Vesterlund et al., 2011). Other studies have demonstrated that SOCS2 is essential for the regulation of GH actions not directly related to somatic growth. For example, SOCS2 can block GH-dependent inhibition of neural stem cell differentiation. Consequently SOCS2KO mice have fewer neurons in the developing cortex, whereas SOCS2 overexpression results in increased neural differentiation. It has also been demonstrated that SOCS2 inhibits intestinal epithelial proliferation (Miller et al., 2004). Recently, we have identified SOCS2 as an important regulator of hepatic homeostasis (lipid and glucose metabolism and inflammation) under conditions of high-fat dietary stress (Zadjali et al., 2012). In addition to GHR down-regulation, other mechanisms are needed to complete inactivation of GH signaling. Since activation of GH-dependent signaling pathways is critically based on protein phosphorylation on tyrosine, serine, or threonine residues, the obvious mechanism for deactivation of this process is the action of protein phosphatases. First, several studies have resulted in the identification of phosphatases which are involved in the specific inactivation of GHR signaling. Second, signal regulatory protein (SIRP)-α, which belongs to a family of ubiquitously expressed transmembrane glycoproteins, negatively regulates GH-activated signaling by inhibition of the phosphorylation of JAK2, STAT5b, STAT3, and ERK1-2 but the physiological relevance of this mechanism is uncertain (Stofega et al., 1998).

### STAT5B, a GH signaling intermediate regulating somatic growth, lipid metabolism, and gender dimorphism

Global expression analysis of GH actions in liver using microarrays clearly indicates that most of the known physiological effects of GH can be explained through its effects on the transcription of specific genes (Flores-Morales et al., 2001; Tollet-Egnell et al., 2001; Waxman and O’Connor, 2006; Ceseña et al., 2007; Vidal et al., 2007). To this end, GH is known to regulate a network of transcription factors that include, among others, nuclear receptors/transcription factors such as HNF (4α, 6, 3β), PPARα, CAR, FXR, SHP, SREBP, CRBP, C/EBPβ, and STAT5b. Based on the analysis of liver transcript profiles from targeted disruption/mutation of signaling components of GHR-signaling pathways or GHR itself, and GH administration to GH-deficient (GHD) mice and rats, the main metabolic process affected by GH status is energy/fuel metabolism, particularly lipid/fat metabolism. These findings in animals together with clinical studies of GH-insensitive subjects have revealed the transcription factor STAT5b is a key GH signaling intermediate for the regulation of target genes associated with several liver physiological processes, including modulation of body growth, cell cycle, and metabolism of lipids, bile acid, steroids, and drugs (Baik et al., 2011). In addition, many transcripts are regulated independently of STAT5b, presumably as a result of GHR-dependent activation of ERK, Src, and PI3K signaling pathways.

### Somatic growth

Growth hormone is predominantly linked to postnatal growth (Butler and Le Roith, 2001). Liver is a major target tissue of GH and the principal source of circulating IGF-I and the GH-dependent transcription of IGF-I is directly regulated by STAT5 binding in IGF-I gene promoter and enhancers (Ohlsson et al., 2009; Rotwein, 2012). Thus, both IGF-I and its transcriptional regulator STAT5 have key roles in mediating the actions of GH on body growth. Importantly, intermittent (male pattern) GH administration to rodents is a more potent stimulus of body growth rate, IGF-I expression, and STAT5b nuclear translocation in liver than is continuous (female pattern) administration. This supports the notion that larger body growth in male compared with female rodents could be due to more effective stimulation of IGF-I and STAT5b mediated transcription. IGF-I proteins are also induced by GH in many tissues and local induction of IGF-I in chondrocytes plays an important role in longitudinal growth. GH is, however, more effective than IGF-I because GH exerts additional growth-promoting actions independent of IGF-I (Lupu et al., 2001). Importantly, global disruption of STAT5b in mice causes loss of sexually dimorphic growth characteristics, so that the affected males reduced their size to female size while female mice appeared unaffected (Udy et al., 1997). Parallel observations were made with circulating IGF-I, which is reduced by 30–50% in affected male mice, but not in females. However, combined disruption of STAT5a/b significantly reduced body weight gain in females and suppressed body growth more than in STAT5b null male mice, approaching that observed either GH or the GHR deficient mice (Rowland et al., 2005). These studies demonstrated that STAT5b is important for male-specific body growth, whereas STAT5a regulates body growth in both sexes. Experiments in SOCS2KO mice also support that STAT5b is critical for GH-regulated somatic growth in mammals (Greenhalgh et al., 2005). Importantly, SOCS2KO mice have enhanced growth whereas combined STAT5bKO and SOCS2KO mice do not, a demonstration that STAT5b is needed for the excess of body growth observed in SOCS2KO mice. In addition to endocrine actions, paracrine involvement of STAT5a/b in the effects of GH on muscle is also evident in the loss of muscle IGF-I transcripts and mass seen with muscle-specific deletion of Stat5a/b (Klover and Hennighausen, 2007). As mentioned above, the growth of female STAT5bKO mice is normal whereas postnatal growth in female GHR-deleted mice is profoundly retarded. These data suggest that in addition to STAT5b, other transcriptions factors are related with growth. This is exemplified by the glucocorticoid receptor (GR) which is a critical co-activator of STAT5b in liver: near 25% of STAT5b-regulated hepatic genes are regulated by a GR-STAT5b transcriptional complex (Mueller et al., 2012). Importantly, these STAT5b and GR co-regulated transcripts were preferentially enriched in functional groups related to growth and maturation (i.e., IGF-I). Moreover, both direct and indirect interactions between ER and STAT5 should be added to the list of mechanisms regulated by nuclear receptors that modulate GH-dependent transcription (Bjornstrom and Sjoberg, 2005).

### Lipid and glucose metabolism

Physiological effects of GH extend beyond the stimulation of somatic growth. These include anabolic effects on protein synthesis and the regulation of lipid and glucose metabolisms throughout life (LeRoith and Yakar, 2007). The key physiological function of GH is the promotion of protein synthesis and inhibition of protein degradation in muscle, bone, and other large organs, inhibiting the catabolism of glucose and aminoacids by promoting the utilization of lipids as energy source. These systemic effects of GH are achieved through inhibition of insulin actions and the promotion of FFA mobilization from adipose tissue and liver (Lichanska and Waters, 2008; Vijayakumar et al., 2010). The mechanisms of GH actions on lipid metabolism are complex and involve both transcriptional and acute changes in catalytic enzyme activities (Lichanska and Waters, 2008). It is well established that human GH is a lipolytic hormone in adipose tissue. One of the mechanisms by which GH leads to lipolytic effects involve increased expression of β3-adrenergic receptor in adipocytes followed by activation of hormone sensitive lipase. Additional effects include uncoupling of the electron transport chain which enhances mitochondrial heat generation at the expense of energy production from ATP. Long-term administration of GH includes a decrease in fat deposition and an increase in fat mobilization, increasing circulating FFA and glycerol levels. GH reduces fat mass, particularly in individuals who have accumulated excess fat during periods of GH deficiency. Obesity is clinically evident in GHD patients, a decline in GH levels correlates with age-related adiposity (Corpas et al., 1993) and lack of GH or GH signaling induces obesity earlier in mice (Lichanska and Waters, 2008). GHD in adulthood causes a syndrome characterized by increased visceral adiposity, decreased muscle mass, metabolic disturbances, and increase mortality associated with cancer or vascular complications. This syndrome closely resembles the metabolic syndrome and can be ameliorated by GH replacement (LeRoith and Yakar, 2007; Lichanska and Waters, 2008). In the skeletal muscle, GH can induce triglyceride (TG) uptake by increasing lipoprotein lipase (LPL) activity and thereby promoting lipid to be stored or utilized via lipid oxidation. Several factors such as nutrition, exercise, and sex-steroid hormone status can modify GH-induced TG storage and lipid oxidation in skeletal muscle. In the liver, GH exerts a complex regulation of lipid metabolism. GH can induce TG uptake, by increasing LPL and/or hepatic lipase expression, and TG synthesis and secretion. Data indicate that an ineffective GHR-JAK2-STAT5 signaling pathway results in fatty liver in rodents which is due to enhanced lipogenesis, increased FA uptake, and/or reduced TG secretion (Cui et al., 2007; Fan et al., 2009; Barclay et al., 2011; Sos et al., 2011). The fatty liver observed in mice with liver-specific disruption of JAK2 seems to be developed by both GH-dependent increases in fat mobilization from adipocytes, increasing circulating FFA, and increased hepatic uptake of FFA (Sos et al., 2011). In previous studies with GH-treated hypophysectomized rats we demonstrated that GH decreases PPARα expression and lipid oxidation while increasing the expression of genes promoting *de novo* lipid synthesis in liver (Flores-Morales et al., 2001). Additional studies in bovine GH-transgenic (Olsson et al., 2003; Wang et al., 2007) and dwarf (Stauber et al., 2005) mice, have all revealed that GH down-regulates genes involved in lipid oxidation and increases the expression of genes promoting lipogenesis in the liver. In contrast, the ablation of SOCS2 in mice, which increases STAT5 signaling, protects from high-fat diet-induced liver steatosis (Zadjali et al., 2012). The deficiency of GHR-JAK2-STAT5 signaling has also been studied by mutagenesis of GHR in mice, a model that causes severe obesity in mature mice in proportion to loss of STAT5b activity (Lichanska and Waters, 2008). These data have shown that STAT5 regulates several key enzymes or genes otherwise involved in lipid and energy balance and, based on altered transcript expression, several processes have been implicated. For example, up-regulation of some lipogenic genes (e.g., CD36, FAS, PPARγ, PGC1α/β, SCD1) may contribute to increased hepatic lipid storage, steatosis, and adiposity in deficient GHR-JAK2-STAT5 signaling models whereas expression of antilipogenic genes such as FGF21 and INSIG2 are decreased. These data have provided new insights into the long-known anti-adiposity actions of GH and highlighted a key role for STAT5 in these actions. This is supported by original findings that STAT5b-deleted male mice become obese in later life (Udy et al., 1997) and that STAT5b deletion in a mature human was associated with obesity (Vidarsdottir et al., 2006). These findings highlight two physiological aspects of GHR-STAT5 signaling: (1) the anti-obesity actions of GH are enhanced by the pulsatility of GH secretion evident in males because of pulsatile STAT5 activation and (2) despite normal plasma FFA and minimal adiposity, absent GHR activation lead to hepatic steatosis because of reduced STAT5 activation, which prevents this pathology (Lichanska and Waters, 2008).

In liver, GH has a stimulatory effect on glucose production which may be a result of its antagonism of insulin action leading to hepatic/systemic insulin resistance (Vijayakumar et al., 2010). GH increases glucose production by increasing glycogenolysis; however, it has either a stimulatory or no effect on gluconeogenesis. Over-expressing the human GH gene in rat increases basal hepatic glucose uptake and glycogen content (Cho et al., 2006). In contrast, GHD mice (Ames) and the GHRKO mice have improved insulin sensitivity and up-regulated hepatic insulin signaling, suggesting that GH antagonizes insulin signaling locally in the liver (Dominici and Turyn, 2002). GH-induced insulin resistance may be developed by the increased FFA mobilization from adipose tissue which can then affects liver insulin sensitivity, and lead to insulin resistance and up-regulation of the PEPCK and G6Pase. However, the LID mice (i.e., IGF-I specific liver deficient mice) showed a 75% reduction in circulating IGF-I levels, threefold to fourfold increase in circulating GH levels and insulin resistance, without significant increase in circulating FFA levels, arguing for the existence of a local crosstalk between GH and insulin signaling systems within the hepatocyte. Moreover, while crossing LID mice with GH transgenic mice, serum FFA levels were significantly increased and there was an improvement in insulin sensitivity during a hyperinsulinemic-euglycemic clamp due to higher hepatic, adipose tissue, and skeletal muscle glucose uptake (Yakar et al., 2004). This suggests that, in addition to FFA, other factor(s) may also contribute to GH-induced insulin resistance. A candidate is the SOCS family of proteins (e.g., SOCS3 and SOCS2) whose expression is induced by both GH and insulin in the liver (Rico-Bautista et al., 2006). However, we have recently shown that deletion of SOCS2 protects against hepatic steatosis but worsen insulin resistance in high-fat diet-fed mice (Zadjali et al., 2012). Another mechanism by which GH may induce insulin resistance is by increasing the expression of the p85, a regulatory subunit of the PI3K (LeRoith and Yakar, 2007). Finally, given the large homologies between the insulin and IGF-I systems, it is not surprising that IGF-I exerts profound effects on carbohydrate metabolism. Alternatively, IGF-I may enhance insulin sensitivity by suppressing GH release, via negative feedback. Therefore activation of IGF-I signaling adds more complexity for understanding molecular mechanisms involved in GH-induced insulin resistance *in vivo*.

### Gender dimorphism

Sex hormones imprint a sex-dependent pattern of pituitary GH hormone secretion which is a major player in establishing and maintaining the sexual dimorphism of hepatic gene transcription that emerges in rodents at puberty (Mode and Gustafsson, 2006). Genomic and bioinformatic analysis have contributed to solve molecular mechanisms involved in GH-regulated hepatic gene transcription (Flores-Morales et al., 2001; Tollet-Egnell et al., 2004; Ståhlberg et al., 2005; Waxman and O’Connor, 2006; Wauthier et al., 2010). Sex-dependent expression and GH regulation characterizes several families of hepatic genes involved in endo- and xenobiotic metabolism as well as relevant metabolic functions (e.g., lipid metabolism); 20–30% of all hepatic genes have a sex-specific expression pattern in rodents. Most of these hepatic sex differences are explained by the female-specific secretion pattern of GH, through the induction of female-predominant transcripts and suppression of male-predominant. A key player in this scenario is STAT5b. Primary results from experiments with STAT5b null mice indicated that STAT5b is responsible for the masculinization of the male liver (Udy et al., 1997; Waxman and O’Connor, 2006). STAT5b binding sites have been found in the promoter of several sex-differentiated CYP genes in rat (e.g., Cyp2c12, Cyp2c11, Cyp2a2). Conversely, other transcription factors (e.g., HNF6 and HNF3b) are more efficiently activated in female liver or by the continuous GH secretion pattern. HNF4 and HNF3b are relevant transcription factors for regulating genes involved in glucose and lipid metabolism (Wolfrum et al., 2004; Sampath and Ntambi, 2005) and most likely they also contribute to sexual dimorphism. Continuous administration of GH has been shown to increase hepatic expression of transcription factor SREBP-1c and its downstream target genes (Tollet-Egnell et al., 2001), as well as hepatic TG synthesis and VLDL secretion (Sjoberg et al., 1996). As mentioned above, GH actions in liver lead to increased lipogenesis (i.e., induction of SREBP-1c) and decreased lipid oxidation (i.e., inhibition of PPARα), and promoted anabolic growth in peripheral tissues (i.e., muscle, bone) (Flores-Morales et al., 2001; Tollet-Egnell et al., 2004; Ståhlberg et al., 2005). Relevant to this review, estrogens cause opposite effects on lipid and glucose metabolism which represents a relevant point of regulatory interactions between estrogens and GH (see below).

## The Liver, A Physiological Target for Estrogens

Estrogen signaling can be mediated by multiple receptors (Heldring et al., 2007). Most of the known estrogenic effects are mediated via direct interaction of estrogen with the DNA-binding transcription factors, ERα and ERβ. Classical estrogen signaling occurs through a direct binding of ER dimers to estrogen responsive elements in the regulatory regions of estrogen target genes followed by activation of the transcriptional machinery at the transcription start site. In addition, estrogen can modulate gene expression by a second mechanism in which ERs interact with other transcription factors, like STAT5, through a process referred to as transcription factor crosstalk. Estrogen may also elicit effects through non-genomic mechanisms, which involve the activation of downstream kinase pathways like PKA, PKC, and MAPK via membrane-localized ERs. An orphan G protein-coupled receptor (GPR)-30 in the cell membrane mediates non-genomic and rapid estrogen signaling. Finally, E2 has a similar affinity for ERα and ERβ and these receptors are activated by a wide range of ligands including selective estrogen receptor modulators (SERMs) (e.g., raloxifene) as well as many other compounds. ERβ is expressed in the ovary, prostate, lung, gastrointestinal tract, bladder, and hematopoietic and the central nervous systems, while ERα is mainly expressed in reproductive tissues, kidney, bone, white adipose tissue, and liver. The liver expresses ERα but almost undetectable levels of ERβ which indicates that specific actions of estrogens in liver can be mimicked by using selective ERα agonists such as propyl-pyrazole-triol (PPT) (Lundholm et al., 2008). Collectively, the above mentioned data indicate that the mechanisms involved in ER signaling are influenced by cell phenotype, the target gene, and the activity or crosstalk with other signaling networks. The liver represents a site where physiologically and therapeutically relevant interactions between estrogens and GH can be developed. Particularly relevant is the interaction of estrogens with GHR-JAK2-STAT5 signaling pathway in the regulation of somatic growth, lipid and glucose metabolism, and “liver sexuality.”

### Somatic growth and body composition

It is well known that sex steroids and GH interact closely to regulate pubertal growth (Kerrigan and Rogol, 1992). Interestingly, loss of ERα (ERαKO), but not ERβ, mediates important effects of estrogen in the skeleton of male mice during growth and maturation (Vidal et al., 2000). A phenotype like to ERαKO mice can be found for aromatase-deficiency in mice or human, which are deficient in estrogens (Riggs et al., 2002). In addition, gender-related differences in body composition are in part mediated by sex steroids modulating the GH-IGF-I axis (LeRoith, 2009; Rogol, 2010; Birzniece et al., 2011). This is supported by the observation of gender differences in body composition emerge at the time of pubertal growth. Furthermore, the efficiency of GH activity is also modulated by estrogens in adulthood. This is exemplified by women being less responsive than men to GH treatment (Burman et al., 1997); GH treatment induces a greater increase in lean mass and decrease in fat mass, or a greater increase in indices of bone turnover and in bone mass, in GHD male compared to female patients. Relevant to GH physiology is the alteration of IGF-I bioavailability by oral administration of pharmacological doses of estrogens [reviewed by Leung et al., 2004]. IGF-I tissue availability and activity are regulated by IGF binding proteins (IGFBPs) (Kaplan and Cohen, 2007; LeRoith and Yakar, 2007; Ohlsson et al., 2009). IGF-I circulates almost entirely as a ternary complex bound to IGFBP-3 and the ALS both of which are strongly GH-regulated in liver. This ternary complex regulates the bioavailability of IGF-I. IGFBP-1 is also a liver-derived protein that binds the small fraction of free IGF-I and attenuates the hypoglycemic effect of the growth factor (Lewitt et al., 1991). In contrast to its suppressive effect on ALS and IGF-I, the oral administration of estrogens increases circulating IGFBP-1. The effect of increased IGFBP-1 can be predicted to reduce further the free fraction of IGF-I, which would be expected to reduce its activity. Interestingly, the activation of GH-STAT5b signaling induces the expression of ALS and IGF-I but inhibits IGFBP-1 (Ono et al., 2007). Therefore, the inhibition of GHR-JAK2-STAT5 signaling pathway in liver (see below), most likely contributes to the effects of estrogens on IGF-I, ALS, and IGFBP-1. Thus, estrogens exert profound effects on liver-derived IGFPBs when administered by the oral route which most likely modify the biological actions of IGF-I. In addition, oral administration of pharmacological doses of estrogen can inhibit GH-regulated metabolic effects (e.g., lipid oxidation, protein synthesis) (Huang and O’Sullivan, 2009). These effects on metabolism and body composition are attenuated by transdermal administration, suggesting that liver is the major site of regulatory control by estrogen.

**Figure 3:**
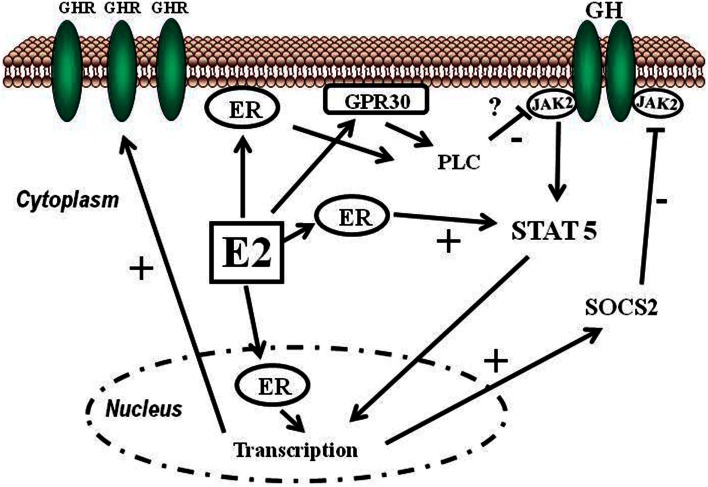
**Schematic representation of signaling pathways activated by E2 and its crosstalk with GH**. Estradiol can regulate GH actions in liver by modulating GH responsiveness which includes changes in hepatic GHR expression and crosstalk with GH-activated JAK2-STAT5 signaling pathway. Estrogens can induce SOCS2 expression which in turn negatively inhibits GHR-JAK2-STAT5 signaling pathway.

Estrogens can modulate GH actions on liver by modulating GH responsiveness, which include changes in hepatic GHR expression and crosstalk with GH-activated JAK2-STAT5 signaling pathway (Leung et al., 2004) (Figure 3). Particularly, E2 can induce SOCS2 and SOCS3 expression which in turn negatively regulates GHR-JAK2-STAT5 signaling pathway leading to reduction in transcriptional activity in liver. Therefore, beside E2 regulation of sex dimorphic pattern of pituitary GH secretion, induction of SOCS expression, and inhibition of JAK2-STAT5 signaling is a very relevant mechanism that, in part, could explain how estrogens directly inhibit the effects of GH in several STAT5-regulated actions (i.e., somatic growth, body composition, metabolism, and gender-related hepatic functions). We have observed that long-term administration of physiological doses of E2 to GHD male rats (hypothyroid) regulates several members of SOCS family by a complex interplay with GH and thyroid hormones (Santana-Farre et al., 2008). Hypothetically, other members of the negative regulators of STAT family may also contribute to estrogen interaction with GH signaling in liver. This is explained by ERα stimulation of PIAS3 expression which binds to and blocks STAT3 DNA-binding activity. Interestingly, E2 activation of ER followed by direct interaction of ER with STAT5 may also inhibit STAT5-dependent transcriptional activity (Faulds et al., 2001; Wang et al., 2004). On the other hand, it has been shown that E2 activation of ERα or ERβ, via non-genomic mechanisms, induces STAT5 (and STAT3)-dependent transcriptional program in endothelial cells (Bjornstrom and Sjoberg, 2005). Overall, these studies have shown a direct interaction between ER and STAT5 signaling and also demonstrate that functional consequence of this crosstalk depends on the precise milieu of the intracellular environment.

### Lipid and glucose metabolism

**Figure 4:**
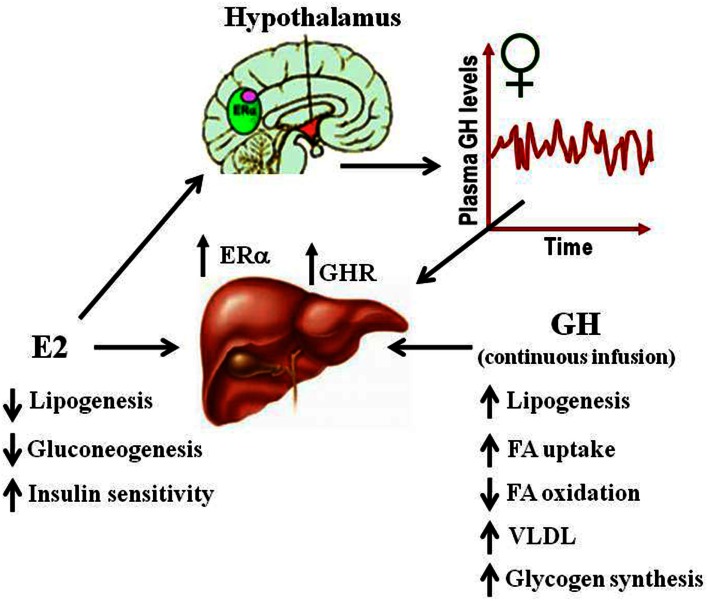
**Physiological effects of E2 and GH on lipid and glucose metabolism in liver**.

Several studies have suggested that E2-mediated signaling can have an important role in the control of lipid and glucose metabolism (Barros and Gustafsson, 2011; Faulds et al., 2012). Studies in both human and rodents suggest that altered levels of estrogen or its receptors can lead to a metabolic syndrome-like phenotype (i.e., insulin resistance, adiposity, dyslipidemia). For example, postmenopausal women are three times more likely to develop metabolic syndrome associated abnormalities than premenopausal women (Eshtiaghi et al., 2010). Furthermore, estrogen/progestin based hormone replacement therapy in postmenopausal women has been shown to lower visceral adipose, fasting serum glucose, and insulin levels (Munoz et al., 2002). Clinical observations in ERα deficient male or with decreased levels of aromatase noted the development of increased body weight, insulin resistance, and hyperinsulinemia (Smith et al., 1994; Jones et al., 2007). Similarly, the ERαKO and the aromatase deficient mice develop insulin resistance, intra-abdominal adiposity, steatosis, and impaired lipid oxidation in liver, which can be reverted by E2 treatment (Heine et al., 2000; Simpson et al., 2005; Jones et al., 2007). The beneficial influence of estrogen in relation to normalizing lipid and glucose homeostasis is also evidenced in ob/ob and high-fat diet-fed mice, models of obesity, and type 2 diabetes. In both models, E2 treatment improves glucose tolerance and insulin sensitivity, and reduce weight in high-fat diet-fed mice (Gao et al., 2006; Bryzgalova et al., 2008). Studies in ERαKO mice have shown that ERα mainly mediates beneficial metabolic effects of estrogens such as anti-lipogenesis, improvement of insulin sensitivity and glucose tolerance, and reduction of adiposity (Barros and Gustafsson, 2011; Faulds et al., 2012). In contrast, ERβKO mice do not exhibit altered insulin sensitivity and/or alterations in body weight. However, some evidence exists that ERβ may be detrimental for the maintenance of regular glucose and lipid homeostasis. In addition to the observations from ERαKO, selective ablations of ERα in the hypothalamic brain region or the hematopoietic/myeloid cells have both been reported to give rise to an increase in body weight and reduced glucose tolerance (Ribas et al., 2011; Xu et al., 2011). Insulin resistance in ERαKO mice is largely localized to the liver, including increased lipid content and hepatic glucose production. Surprisingly, the liver-selective ablation of ERα (LERKO) did not recapitulate the observed ERαKO mice phenotype (Matic et al., 2013). LERKO mice did not increase body weight nor developed glucose intolerance or insulin resistance, even when challenged with a high-fat diet. The authors suggest the presence of unidentified compensatory mechanism/s or that hepatic insulin resistance occurs as a secondary effect upon ablation of E2 signaling in other cell types. Furthermore, treatment of ob/ob mice with the ERα-selective agonist PPT can improve glucose tolerance and insulin sensitivity which supports the critical role of ERα signaling in the control of glucose homeostasis. Estrogenic signaling via GPR-30 has also been implicated in insulin production and glucose homeostasis (Mårtensson et al., 2009). As mentioned above, the absence of E2 or GHR-JAK2-STAT5 signaling causes adiposity and hepatic steatosis which can be ameliorated by E2 (Heine et al., 2000; Simpson et al., 2005; Jones et al., 2007) or GH (LeRoith and Yakar, 2007) replacement, respectively. This suggests that E2 and GH signaling regulate overlapping cellular networks related with physiological control of lipid and glucose metabolism (Figure 4).

### The “liver sexuality,” a phenotype regulated by GH and sex steroids

As mentioned above, sex steroids are physiological regulators of pituitary GH secretion and, indirectly, regulate sex-specific liver physiology (Mode and Gustafsson, 2006). From neonatal period of life, gonadal steroids play a critical role to maintain liver response to GH in adulthood. Neonatal exposure to androgens is crucial and the full response to androgens in adulthood is dependent on neonatal imprinting by androgens. The male characteristic metabolism in liver in adulthood is dependent on continuous androgen exposure. In female rats, gonadectomy has little impact on hepatic steroid metabolism; estrogen treatment, however, feminizes hepatic metabolism in male rats. Genome-wide screens of gene expression have shown that GH- and sex-dependent regulation of hepatic gene expression is not confined to steroid or drug metabolism. Moreover, a number of other hepatic genes have been found to be up- and/or down-regulated by the different patterns of GH or sex-steroid exposure. GH- and sex-dependent hepatic transcripts encoding plasma proteins, enzymes, transcription factors, and receptors involved in the metabolism of proteins, carbohydrates, lipids, or signaling regulation have been identified (Flores-Morales et al., 2001; Ståhlberg et al., 2005; Waxman and Holloway, 2009). A consensus exists that the response to sex-different GH patterns is the major cause of gender dimorphism in liver; however, it is likely that factors other than the sexually dimorphic pattern of GH secretion are behind some sex differences in rat liver. Potential mechanisms that could contribute to “liver sexuality” are the pituitary-independent effects of estrogens through interaction with ERα or GH-JAK2-STAT5 signaling pathway in liver.

## Conclusion

Growth hormone and E2 are critical regulators of body growth and composition, somatic development, metabolism, and gender dimorphism. GH and E2 signaling play a critical role in liver physiology and pathology in both female and male. Physiologically and therapeutically relevant are E2 interactions with GH-regulated endocrine (e.g., IGF-I), metabolic (e.g., lipid and glucose metabolism), and sex-differentiated (e.g., endo- and xenobiotic metabolism) functions in liver. The influence of estrogens is executed at the level of pituitary GH secretion and the regulation of GHR-JAK2-STAT5-SOCS signaling pathway. Therefore, the complex estrogen/GH interplay is relevant because physiological roles that these hormones have in mammals, and the widespread use of estrogen-related compounds. In the general population, the endocrine and metabolic consequences of long-term exposition to estrogens or novel estrogen-related compounds and their influence on the GH axis are largely unknown. Understanding this complex interaction in physiological and pathological states could contribute to prevent health damage and improve clinical management of patients with growth, developmental, and metabolic disorders.

## Conflict of Interest Statement

The authors declare that the research was conducted in the absence of any commercial or financial relationships that could be construed as a potential conflict of interest.
